# Cardiovascular Magnetic Resonance as Pathophysiologic Tool in Diabetes Mellitus

**DOI:** 10.3389/fendo.2021.672302

**Published:** 2021-06-14

**Authors:** Sophie I. Mavrogeni, Flora Bacopoulou, George Markousis-Mavrogenis, Aikaterini Giannakopoulou, Ourania Kariki, Vasiliki Vartela, Genovefa Kolovou, Evangelia Charmandari, George Chrousos

**Affiliations:** ^1^ Department of Cardiology, Onassis Cardiac Surgery Center, Kallithea, Greece; ^2^ University Research Institute of Maternal and Child Health and Precision Medicine, and UNESCO Chair on Adolescent Health Care, National and Kapodistrian University of Athens, Aghia Sophia Children’s Hospital, Athens, Greece; ^3^ Department of Pediatric Cardiology, Aghia Sophia Children’s Hospital, Athens, Greece; ^4^ Division of Endocrinology, Metabolism and Diabetes, First Department of Pediatrics, School of Medicine, National and Kapodistrian University of Athens, Aghia Sophia Children’s Hospital, Athens, Greece; ^5^ Division of Endocrinology and Metabolism, Center for Clinical, Experimental Surgery and Translational Research, Biomedical Research Foundation of the Academy of Athens, Athens, Greece

**Keywords:** cardiovascular, magnetic resonance, coronary artery disease, heart failure, myocardial fibrosis, diabetes, cardiac MRI, cardiac function

## Abstract

Diabetes mellitus can independently contribute to cardiovascular disease and represents a severe risk factor for premature development of cardiovascular disease. A three-fold higher mortality than the general population has been observed in type 1 diabetes mellitus whereas a two- to four-fold increased probability to develop cardiovascular disease has been observed in type 2 diabetes mellitus. Cardiovascular magnetic resonance, a non-radiative modality, is superior to all other modalities in detecting myocardial infarction. The main cardiovascular magnetic resonance sequences used include a) balanced steady-state free precession (bSSFP) for function evaluation; b) T2-W for oedema detection; c) T1 W for ischemia detection during adenosine stress; and d) late gadolinium enhanced T1-W images (LGE), evaluated 15 min after injection of paramagnetic contrast agent gadolinium, which permit the diagnosis of replacement fibrosis, which appears white in the middle of suppressed, nulled myocardium. Although LGE is the technique of choice for diagnosis of replacement fibrosis, it cannot assess diffuse myocardial fibrosis. The application of T1 mapping (native or pre contrast and post contrast) allows identification of diffuse myocardial fibrosis, which is not detectable my other means. Native T1 and Contrast-enhanced T1 mapping are involved in the extracellular volume fraction (ECV) calculation. Recently, 1H-cardiovascular magnetic resonance spectroscopy has been applied to calculate the amount of myocardial triglycerides, but at the moment it is not part of the routine assessment of diabetes mellitus. The multifaceted nature of cardiovascular magnetic resonance has the great potential of concurrent evaluation of function and myocardial ischemia/fibrosis in the same examination and represents an indispensable tool for accurate diagnosis of cardiovascular disease in diabetes mellitus.

## Introduction

The clinical implications of diabetes mellitus (DM) on the cardiovascular system are profound with serious consequences that are reflected in patients’ survival worldwide. Insulin resistance can directly contribute to cardiovascular disease (CVD) (1). Furthermore, asymptomatic patients with DM demonstrate impaired myocardial perfusion rate index (MPRI), compared with normal volunteers (2). Cardiovascular magnetic resonance (CMR) can detect occult myocardial lesions and reduced microvascular perfusion in patients with early type 2 DM (T2DM) (3).

Patients with DM have increased CVD morbidity/mortality as a result of various pathophysiologic changes, including epicardial coronary artery disease (CAD) leading to myocardial infarction (MI), microvascular CAD, endothelial dysfunction, cardiac remodeling due to diffuse myocardial fibrosis, fatty myocardial infiltration and diastolic dysfunction leading to heart failure (HF). Finally, peripheral vascular disease, involving carotid arteries and brain vasculature, may cause claudication and stroke, respectively (4).

## Cardiovascular Disease in DM

Three-fold higher mortality has been identified in patients with type 1 diabetes mellitus (T1DM) compared to controls, due to premature atherosclerosis in both men and women (5). In this population, CVD events appear a decade earlier, in comparison with nondiabetic subjects (6). A recent meta-analysis found that the CVD mortality rate was 5.7 for men and 11.3 for women with T1DM (7, 8). Recent findings showed a 10-fold increased risk of CVD mortality in T1DM depending on glycemic levels (9). Furthermore, coronary artery calcifications were identified in >70% of men and >50% of women over 45 years with T1DM (10). Adequate management of CVD risk factors led to a 29% reduction in the cardiac death risk over next 10 years (11). This resulted to a CVD risk of 2.3 in men and 3.0 in women (12). The excess risk in women with T1DM cannot be attributed to the usual CVD risk factors (13) and supports that women with T1DM are not protected against CVD (14). It is important to notice that male and female patients with T1DM have the same risk of CVD (15).

CAD and myocardial infarction (MI) are two to four times more common in patients with T2DM (16). Therefore, T2DM is an independent factor for stroke and CVD (17), with almost 70% of T2DM patients dying at age < 65 years due to CVD (16). T2DM patients without CAD are at the same risk with patients with previous MI (18). Classic CVD risk factors can additionally augment the CVD risk in T2DM. It is well documented that insulin resistance and hyperglycemia lead to low-grade inflammation triggering endothelial dysfunction (19–21). Blood inflammatory indices together with increased platelets activity are the most important causative factors (22, 23).

## Non-Invasive Cardiovascular Imaging in DM

Various noninvasive imaging modalities have been applied to diagnose early cardiovascular involvement in DM. Among them, echocardiography is the cornerstone modality applied in clinical practice, because it can be used for bedside evaluation, has low cost, is radiation free, and is widely available. However, echocardiography has some serious limitations, including dependency of operator and acoustic window, low reproducibility, and lack to provide information about tissue characteristics. Additionally, it is unable to differentiate between epicardial and microvascular CAD and cannot detect small MIs that do not cause significant wall motion abnormalities (4). Furthermore, exercise echocardiography, using dobutamine as a pharmacologic stress factor, is often suboptimal in T2DM, due obesity, which usually coexists with DM (4).

SPECT imaging is the most commonly used modality to assess myocardial ischemia in DM. However, in DM patients, SPECT has some serious limitations. Its spatial resolution of ≈7 mm × 9 mm does not allow detection of subendocardial ischemia. SPECT relies on regional differences in myocardial blood flow and, therefore, is less accurate in patients with triple vessel disease and balanced ischemia, diffuse microvascular dysfunction and non-transmural MI (4). Furthermore, obese patients usually have lower signal in the inferolateral wall that leads to false-positive results. Despite these limitations, a systematic analysis of SPECT studies showed that in DM patients with and without symptoms, a normal SPECT indicates an annual event rate for MI/cardiac death around 1.9% (24). Finally, SPECT uses radioactive tracers that are not indicated in the young DM patients with rapid disease progression and atypical presentations, who need repeated re-evaluation scans (4).

Computed coronary angiography (CTA) can play a role in assessing DM patients, and there is a great debate regarding its use in asymptomatic patients and the value of calcium screening as a gatekeeper for further ischemia testing (25). However, data from the FACTOR 64 study showed that in DM patients with or without symptoms, CTA does not improve their management or outcome (26).

## Which is the Place of CMR in DM Evaluation?

Cardiovascular Magnetic Resonance is a non-radiative modality, with high spatial resolution (0.3-1mm) capable to provide details regarding both function and tissue characteristics in the same study (27). CMR is superior to all other modalities in detecting MI (4). The ICELAND MI study revealed an incidence of 27% MI in the general population between 67 and 93 years and 32% in patients with DM (27). Importantly, the number of unrecognized MIs was high in both groups (17% and 21%), demonstrating significant underestimation of MI in DM patients. It should be noticed that unrecognized MIs had the same negative impact on patients’ prognosis with known MIs, despite their smaller size. Similarly, other investigators found evidence of MI in 28% of DM patients, and this was the best prognostic factor of future adverse events (28). Turkbey et al. studied 1,017 patients with T1DM (741 using Gd contrast agent, approximately 49 years, DM duration equal to 22 years) and found increased left ventricular (LV) mass with reduced end-diastolic volumes. These parameters were related to CVD risk factors, HbA1c and macroalbuminuria (29). In 4.3% of DM patients, an MI was found by CMR in contrast to only 1.4% by clinical evaluation. Additionally, Rijzewijk et al. found a strong correlation between myocardial steatosis and diastolic abnormalities in DM patients (30). Finally, Heydari et al. evaluated the utility of CMR first-pass perfusion imaging using vasodilatory stress with adenosine or regadenoson for risk classification of DM patients. After examination of 173 symptomatic patients with DM using CMR, and follow-up over 2.9 ± 2.5 years, they found that inducible myocardial ischemia, defined as at least one positive segment of more than one voxel thickness lasting for at least three heartbeats, was the strongest predictor of outcome. Patients with DM with neither ischemia nor MI had a 0.5% event rate for cardiac death or MI, those with no inducible ischemia 1.4% and those with inducible ischemia 8.2% per year. We should also emphasize that the DM duration did not correlate with the prevalence of ischemia (31).

## CMR Techniques To Assess Cardiovascular Disease In Diabetes Mellitus

Cardiovascular sequelae constitute a substantial burden on the treatment of DM at both the patient and population levels (32). CMR can provide non-invasive, highly reproducible, radiation-free information about the status of all cardiac tissues (7). The basic pulse sequences to succeed this target include (33):

1. **Balanced steady-state free precession (bSSFP).** bSSFP is increasingly important to assess myocardial function. It is also the ideal approach to evaluate cardiac anatomy/mass/wall motion/biventricular/atrial function and cardiac remodeling (**Figure 1**) (34).

**Figure 1:**
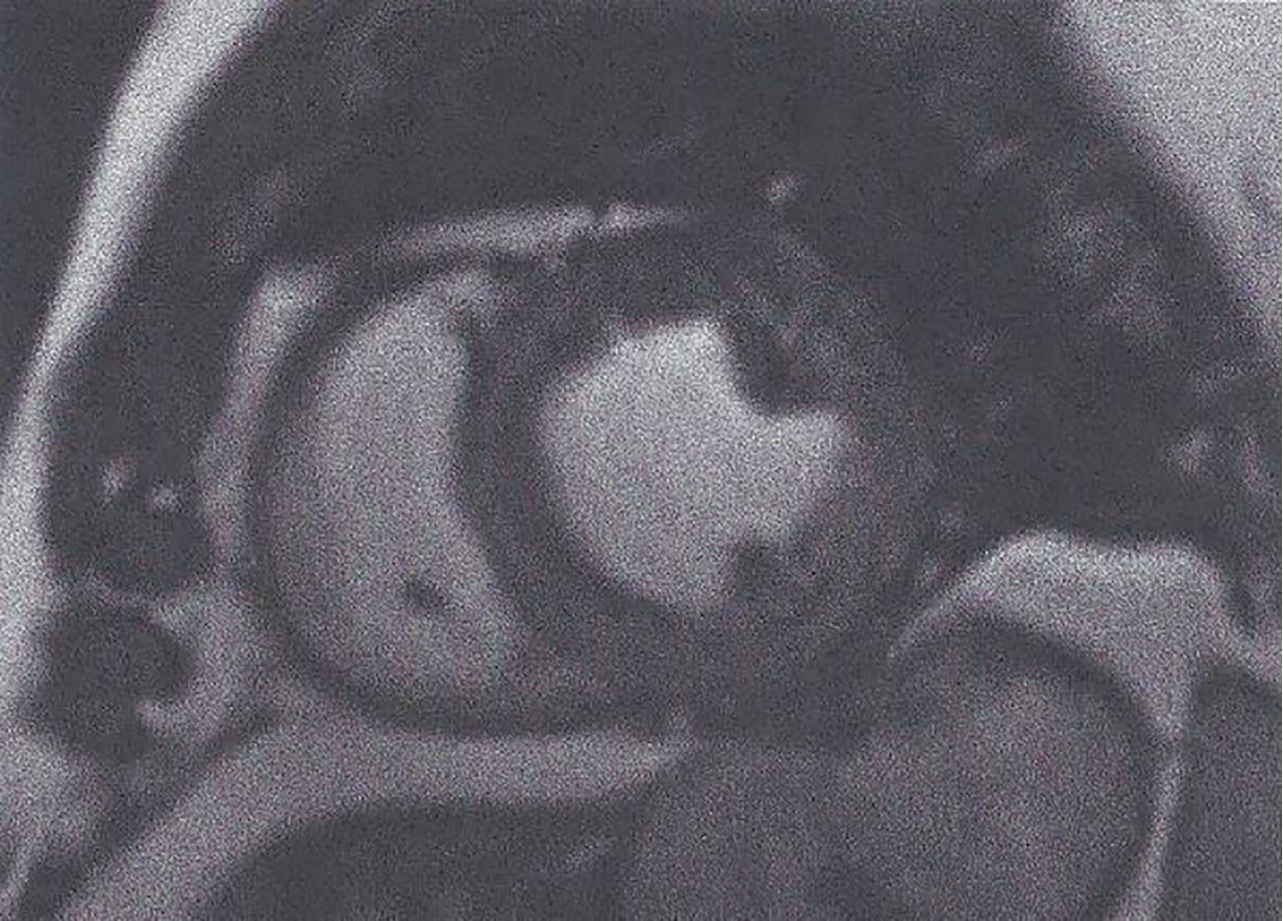
Short axis SSFP image used for myocardial function assessment. This figure is original and based on data from Mavrogeni et al.
2. **T2- weighted images (T2-W).** These images are produced due to increased water amount taken place during oedema (35, 36). Oedema is the acute reaction of myocardium to any kind of damage either ischemic (myocardial infarction) or inflammatory (any type of myocarditis). It may be localized or diffuse, subendocardial, or transmural following the territory of coronary arteries as in CAD (**Figure 2**) and subepicardial or intramyocardial as in myocarditis (**Figure 3**).

**Figure 2:**
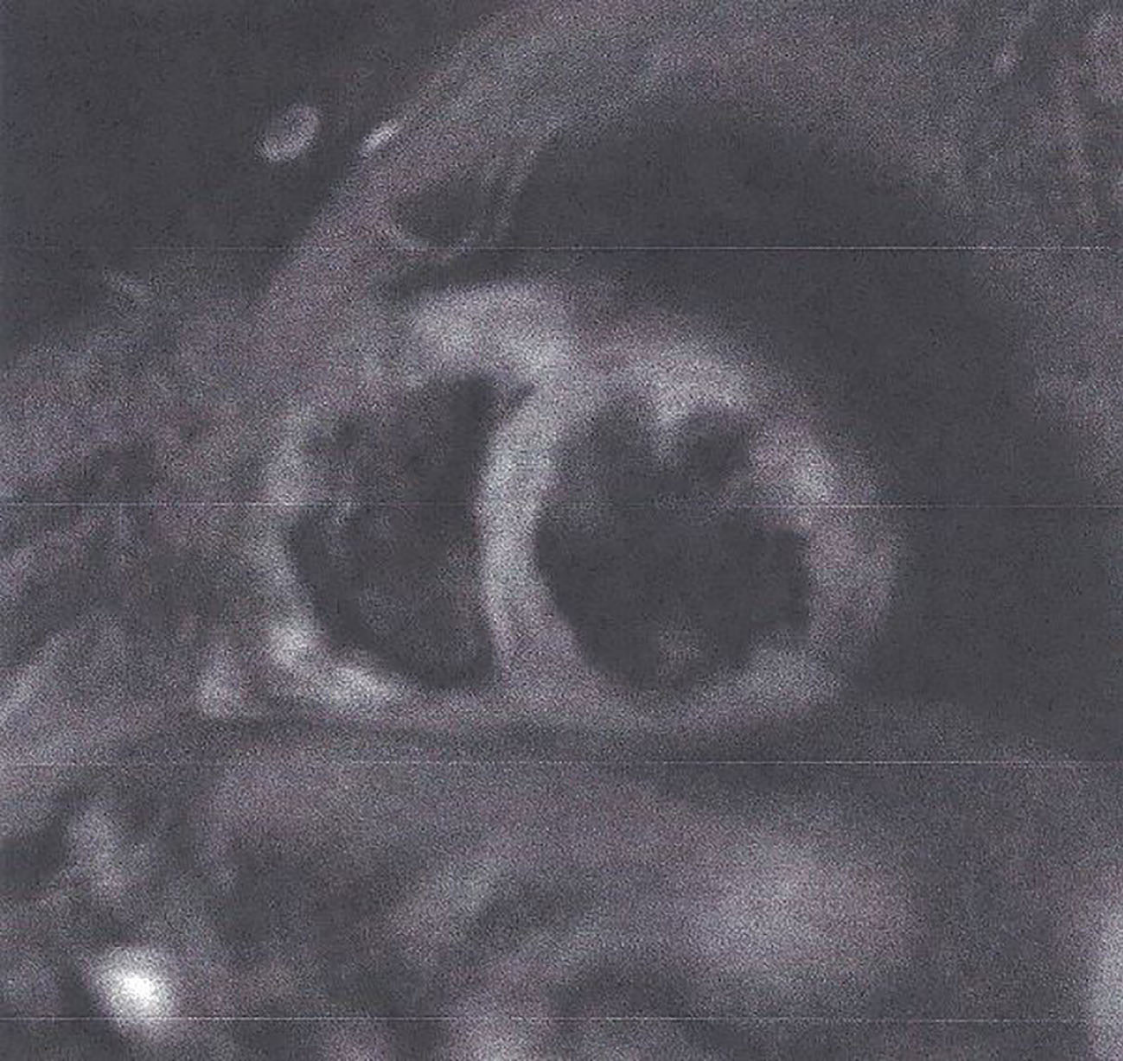
STIR T2 image showing transmural oedema in myocardial infarction. This figure is original and based on data from Mavrogeni et al.

**Figure 3:**
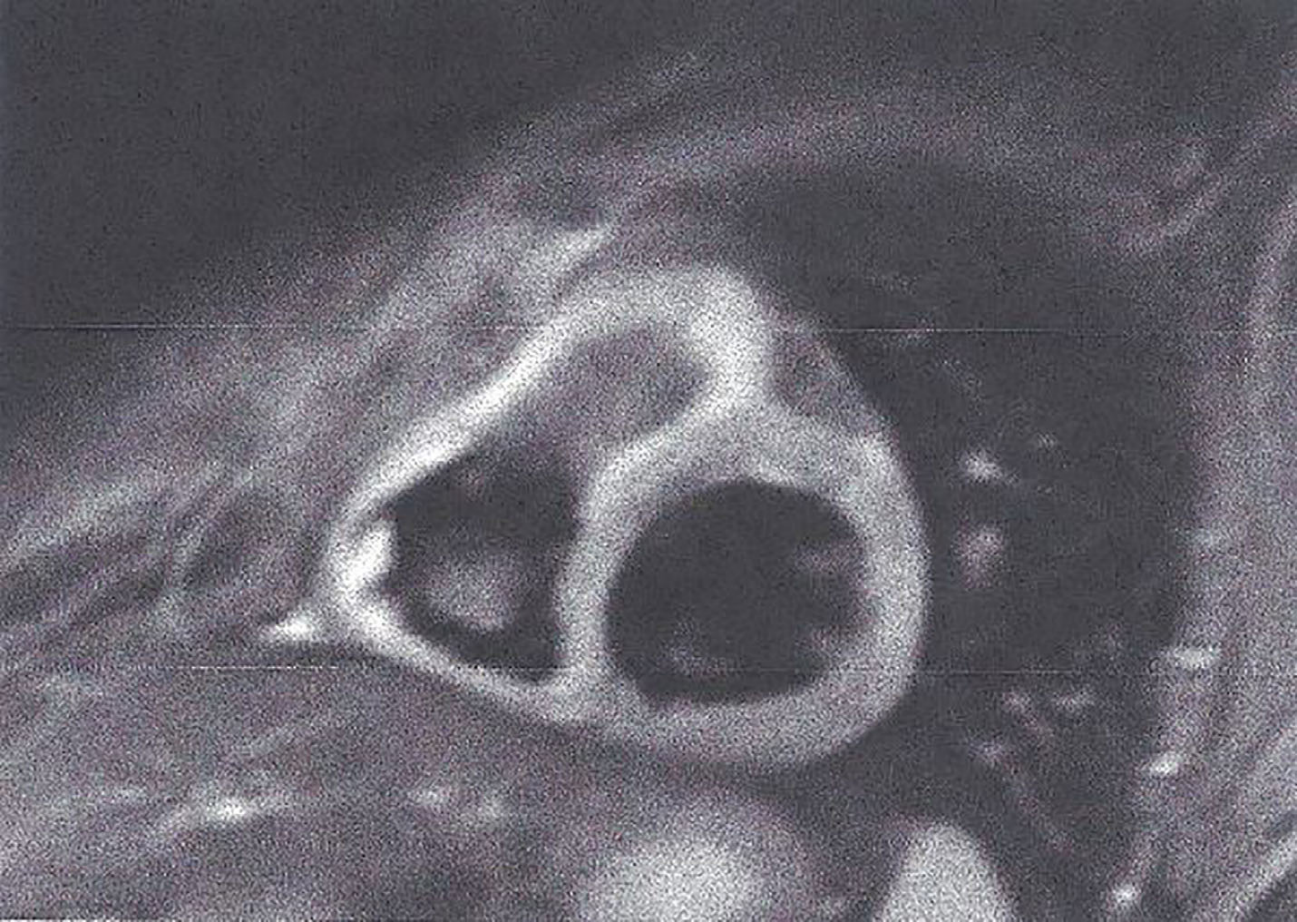
STIR T2 image showing subepicardial oedema in myocarditis. This figure is original and based on data from Mavrogeni et al.
  Oedema presents high intensity on short tau inversion recovery (STIR) images. However, these images may present various limitations (37), including low contrast between healthy/edematous areas, dependency on field homogeneity, blurring due to cardiac motion, subendocardial slow flow hyperintensity, and subjective differences in visual interpretation (37, 38).
  To overcome the STIR limitations, T2 mapping has been developed, based on the transverse relaxation time of each voxel. At 1.5 T, the normal myocardium T2 value was measured at 52 ± 3 ms by Giri et al. in 14 healthy volunteers and at 55 ± 5 ms in 73 healthy volunteers by Wassmuth et al. and were related to body surface area (BSA) or heart rhythm and had excellent reproducibility (39, 40).
3. **T1-weighted images (T1-W).** The T1 relaxation time, an index of how quickly the spins return to equilibrium after a radiofrequency (RF) pulse, is a main parameter of tissue contrast (41). T1-W without contrast agent is ideal for anatomy evaluation. Late gadolinium enhanced T1-W images (LGE), assessed 15 min after gadolinium, permit the detection of myocardial fibrotic areas, which appear as bright in a nulled, black myocardium “bright is dead” (**Figure 4**) (32). According to the type and location of LGE, it is attributed to CAD, if the lesion follows the distribution of coronary arteries. In contrast, subepicardial or patchy LGE usually in the inferolateral wall is characteristic of any kind of myocarditis. This technique performs equally well in the diagnosis of both acute and chronic myocardial scar (32).

**Figure 4:**
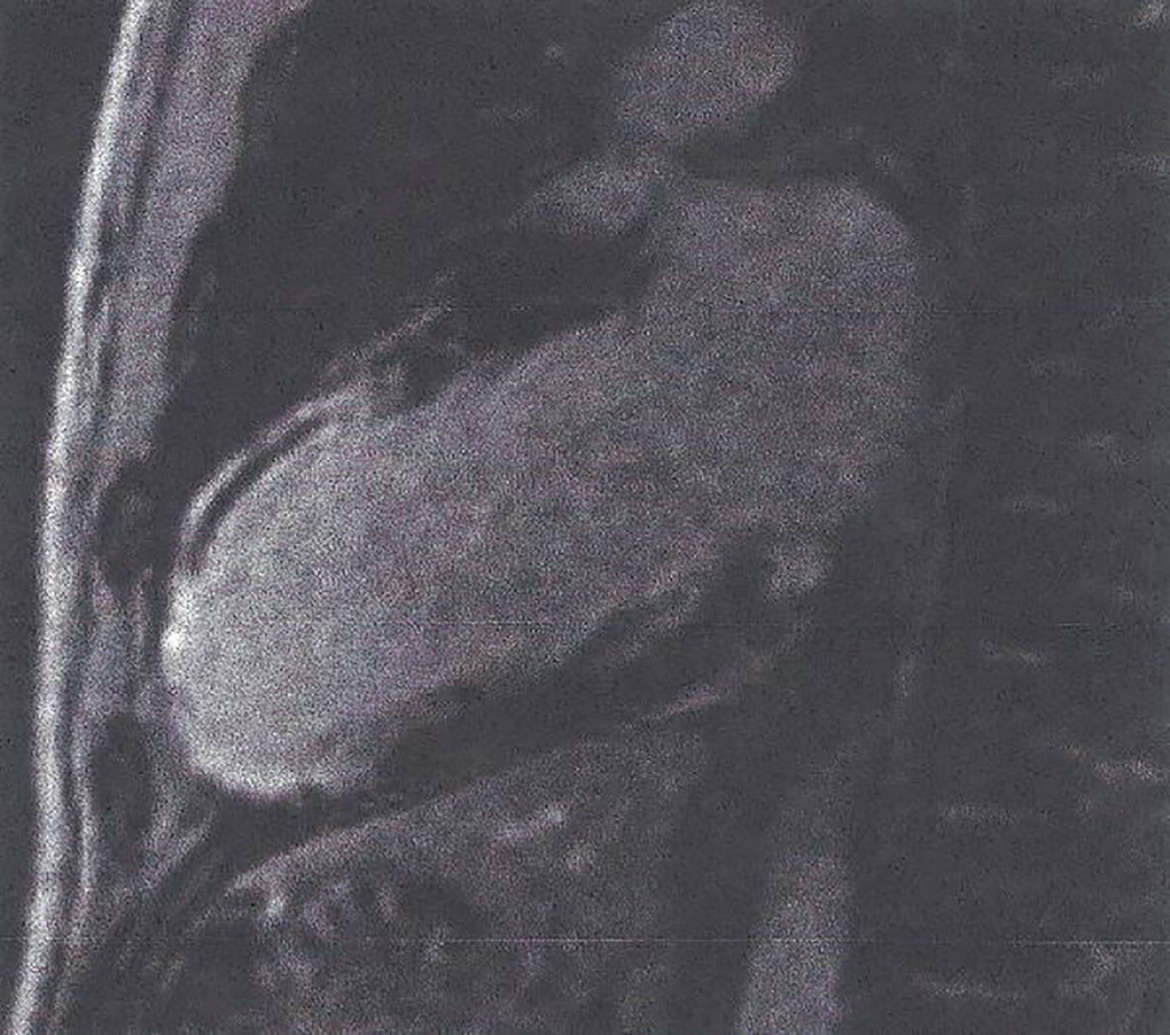
LGE image showing extensive myocardial infarction in anterior wall (bright area) with microvascular obstruction (dark area within the white area of scar) in a patient with T2DM. This figure is original and based on data from Mavrogeni et al.
  T1-W after pharmacologic stress using adenosine or regadenoson and bolus injection of paramagnetic contrast agent can provide accurate, reproducible information about myocardial perfusion during stress (**Figure 5**) (41). This approach has independent prognostic utility and can re-classified CVD risk in DM patients, referred for ischemic assessment (31). Furthermore, almost 1/4 of asymptomatic DM patients with Framingham risk ≥ 20% had occult CAD and reduced global MPRI, compared with normal subjects (42).

**Figure 5:**
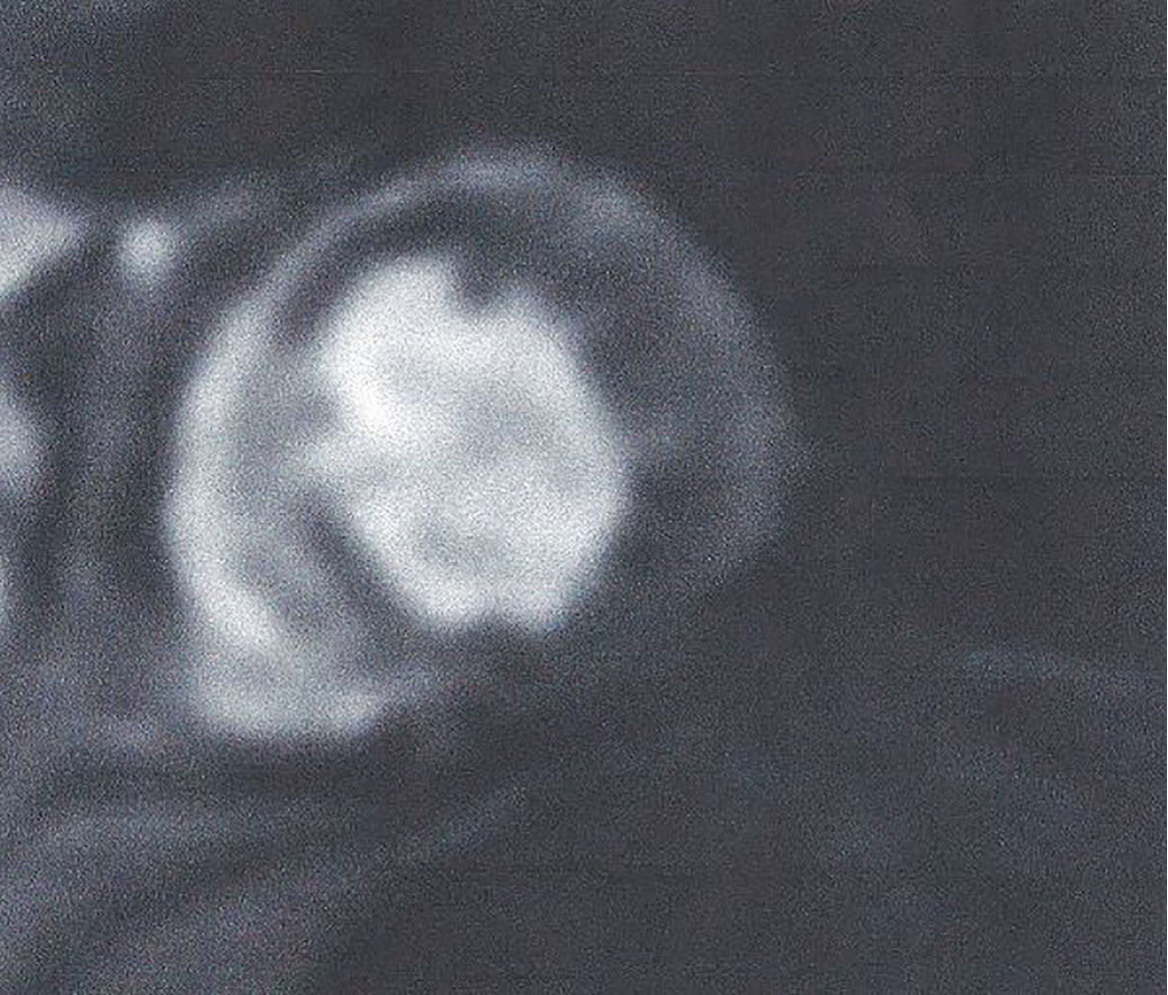
Adenosine stress CMR image showing extensive perfusion defect in anterior and inferolateral wall of LV (dark area) in a patient with T2DM. This figure is original and based on data from Mavrogeni et al.
4. **Parametric imaging (T1, T2 imaging, ECV).** Although LGE is well validated as the technique of choice for the detection of replacement fibrosis, it has serious limitations in the assessment of diffuse myocardial fibrosis, because it is based on the signal intensity differences between fibrotic and normal myocardium to provide image contrast. To overcome this problem, a CMR imaging technique called T1 mapping has been created. T1 mapping identifies early myocardial fibrosis, which is undetectable using the current blood biomarkers (43). Normal values of T1 mapping in 1.5 T are 995.8 ± 30.9 ms and 1,183.8 ± 37.5 ms at 3T (44) (**Figure 6**).

**Figure 6:**
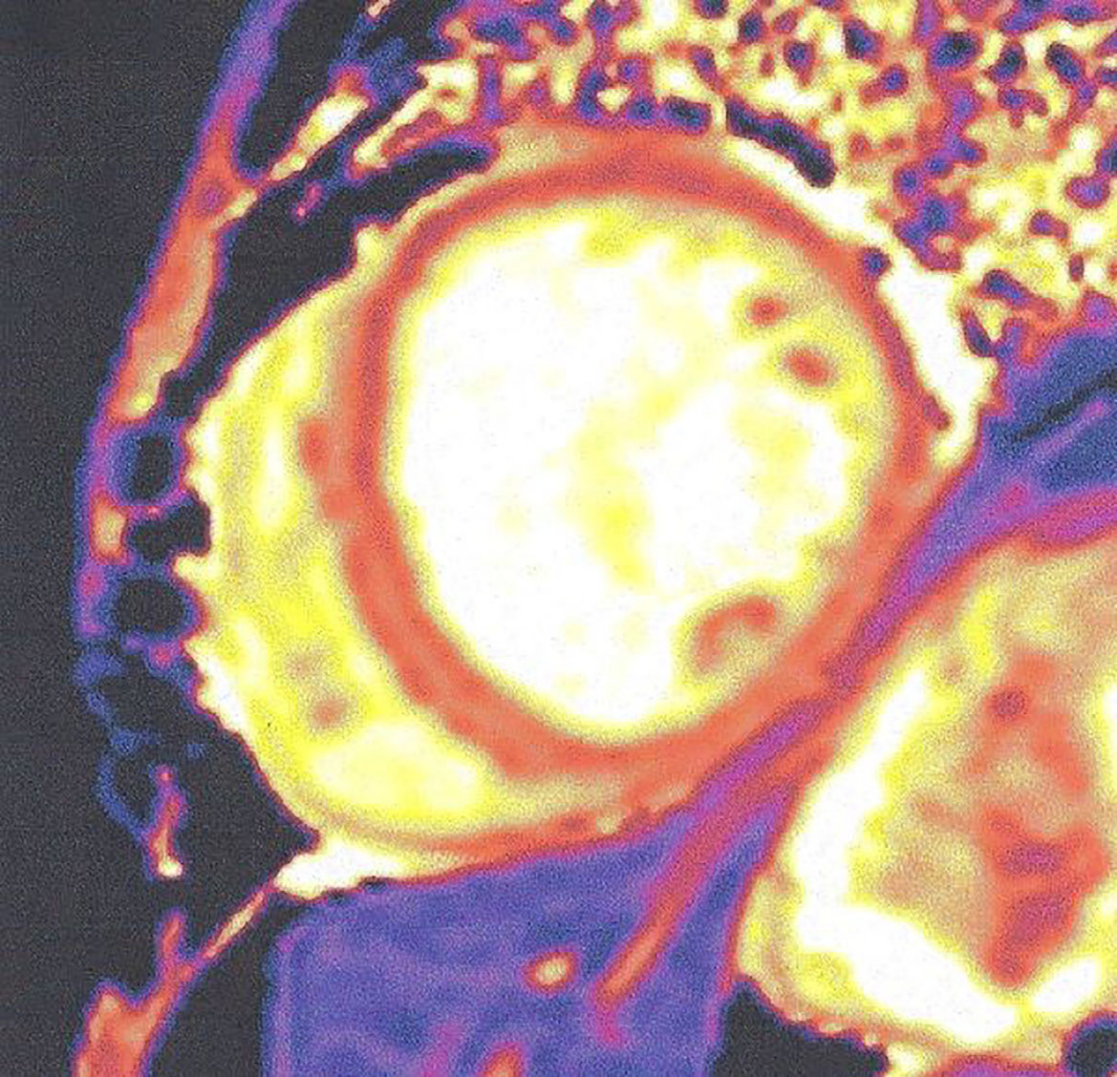
Increased native T1 mapping in a patient with diabetic cardiomyopathy. This figure is original and based on data from Mavrogeni et al.

- Contrast-enhanced T1 mapping is used for the extracellular volume fraction (ECV) calculation together with native T1 mapping. The estimation of the ECV is an index of inter-and extra-cellular area and measurement of myocardial/blood T1 before/after gadolinium together with hematocrit is needed, with hematocrit representing the cellular fraction of blood.
- Normal ECV values equal to 25.3 ± 3.5% were found in healthy subjects, examined at 1.5T scanners (45). With the exception of amyloidosis, an increased ECV is the result of collagen presence, as in case of systemic sclerosis and other cardiomyopathies (46). Low ECV was found in thrombus and lipomatous metaplasia and can be calculated either from T1 mapping images or directly visualized on ECV maps images (46).

Compared to native T1 relaxation times, contrast-enhanced T1 values are depended on various parameters, such as contrast dose, time between contrast injection, and T1 imaging and renal function and therefore are more variable. ECV, on the other hand, is a physiologic index derived from the ratio of T1 values. Therefore, ECV is a more reproducible index in different magnetic fields, acquisition techniques, and vendors than both native/post-contrast T1 (46). Furthermore, ECV has excellent correlation with histologic assessment of the collagen than T1 mapping (47).

Using this approach, it was proven that elevated ECV is an independent factor of mortality in DM representing a novel non-invasive biomarker to evaluate the severity of diabetic heart disease (48). Additionally, DM was found to have increased T1, T2 mapping and ECV, compared to controls, independently of LGE (49). Furthermore, native T1 mapping is sensitive to myocardial infarcts and scarring (16) and allows the follow-up of longitudinal changes during treatment trials (16). Recently, adenosine stress and rest T1 mapping detected ocult abnormalities of the microvascular circulation, without the use of gadolinium, providing diagnostic information for early therapeutic intervention (50). Finally, patients with pre-diabetes or DM and preserved LVEF had increased LV remodeling, suggesting early alterations during the disease process (51). The CMR techniques used for myocardial evaluation of DM are presented in **Table 1**.

**Table 1:** CMR sequences for myocardial evaluation in patients with DM.

Pulse sequence	Myocardial characteristic assessed	Type of study	Cardiac disease in DM
Steady-state free precession (SSFP)	Function of LV-RV	Clinical	LV-RV dysfunction
	Wall motion/thickness		LV hypertrophy
	Myocardial mass		
Inversion recovery for Late Gadolinium Enhancement (LGE)	Replacement fibrosis due to MI	Clinical	Myocarditis
	Myocarditis		Myocardial infarction
	Infiltrative disease		Infiltrative disease
First-pass T1 imaging using stress	Myocardial stress perfusion	Clinical	Myocardial ischemia due to micro-macro- CAD
T1 mapping/ECV	Diffuse myocardial fibrosis	Clinical	Extent and severity of diffuse myocardial fibrosis
1H and 31P CMR spectroscopy	Cardiac triglycerides and cardiac energetics	Experimental	Pre-clinical detection of myocardial involvement

## Advanced CMR Techniques Used in DM

CMR found a correlation between myocardial lipid accumulation and LV diastolic dysfunction, independently of age, blood pressure, and obesity in T2DM (30) and improvement after anti-diabetic treatment (52, 53). Ng et al. described an association between CMR assessed myocardial triglycerides and longitudinal strain measured using echocardiography, suggesting a subclinical effect of lipids on myocardial function in T2DM (54). In another study by Korosoglou et al. evaluated strain-encoded CMR, 1H-CMR spectroscopy for triglycerides quantification and adenosine stress-CMR for myocardial perfusion reserve and found that only myocardial triglycerides were correlated with LV diastolic dysfunction in T2DM. This correlation persisted even after adjustment for demographics and clinical parameters (55).

CMR has been also used to evaluate the effect of treatment. A randomized study to pioglitazone, metformin or placebo for 24 weeks in 78 T2DM men without CVD showed no effect of treatment on myocardial lipids and energetic, measured by 1H and 31P CMR spectroscopy, respectively (56). Similar findings showed the lack of rosiglitazone effect on myocardial lipids using 1H-CMR spectroscopy (57). It is also important to mention that the prolonged caloric restriction in T2DM decreases the myocardial triglycerides leading to amelioration in LV diastolic function (52). Finally, increased myocardial lipid amount was found in patients with impaired glucose tolerance and normal LVEF (52).

In a study using cardiac phosphocreatine-to-ATP ratio, it was found that this index was lower in T2DM patients compared with controls (58). Furthermore, this parameter was correlated with LV diastolic dysfunction (59). Finally, T2DM evaluation using positron emission tomography (PET) and CMR reported a correlation between hepatic triglycerides, measured using 1H-MRI and impaired cardiac perfusion, function, and high-energy phosphate metabolism (60). Furthermore, 31P-CMR assessment in T1DM has shown that the presence of abnormal cardiac high-energy phosphate metabolism is independent of myocardial perfusion and DM duration (61). The capability of these techniques to evaluate the cellular phosphate metabolism and to quantify the myocardial phosphate may be of crucial significance in the validation of novel treatments in DM. However, these exciting applications at the moment are not available in the clinical practice.

Finally, water/fat separation CMR sequences, as well as ^1^H-MRS, have provided valuable insight into the relationships between epicardial adiposity, myocardial fat content, and obesity-related cardiac disease. Whether increased epicardial adiposity in healthy subjects can lead to myocardial fat loading and subclinical impairment of myocardial function is an issue of great interest, with important clinical implications for obesity-related cardiac disease. However, further studies are needed to understand whether quantification of epicardial adiposity and myocardial fat content in normal hearts have any diagnostic, prognostic or therapeutic impact to reverse established myocardial disease in humans (62).

## Cardiovascular Magnetic Resonance: Pro and Contra

Within the significant advantages of CMR are included the lack of radiation, the great reproducibility of the measurements and the capability of performing tissue characterization, independently of blood biomarkers. Its high spatial resolution is of great value in the accurate measurement of myocardial mass, volumes, and biventricular ejection fraction. Furthermore, it is the most reliable modality for the identification of ischemia, fibrosis, edema, and inflammation. However, important disadvantages include limited access to MRI system, lack of expertise, and increased examination cost not covered by the re-imbursement system in most countries. Additionally, there are limitations, such as claustrophobia, long time of examination/processing, inability to examine patients with non-CMR compatible devices. Furthermore, non-cooperative patients cannot be examined without sedation and in patients with renal failure the use of contrast agents is restricted, due to the risk of nephrogenic fibrosis. However, the high cost of CMR examination maybe counterbalanced by the advantage of timely diagnosis and treatment of CVD in DM. In a recently published systematic review regarding the CVD cost in DM patients, it was shown that from a population level, CVD costs contributed between 20% and 49% of the total direct costs of treating T2DM. The median annual costs per patient for CVD, coronary artery disease, heart failure, and stroke were, respectively, 112%, 107%, 59%, and 322% higher compared with those for T2DM patients without CVD. On average, treating patients with CVD and T2DM resulted in a cost increase ranging from $3418 to $9705 compared with treating patients with T2DM alone (32). Finally, the EACVI preparatory course to certification in CMR has a cost of 1280 euros for each candidate.

To assess sensitivity/specificity of CMR vs other imaging modalities, the CE-MARC study (63) evaluated 235 women and 393 men with suspected angina using CMR, SPECT and x-ray angiography. In this study, the CMR evaluation, including adenosine stress/rest perfusion, cine imaging, late gadolinium enhancement, and magnetic resonance coronary angiography, found that the sensitivity in women and men was similar (88.7% versus 85.6%; P=0.57), as was the specificity (83.5% versus 82.8%; P=0.86). For SPECT, the sensitivity was significantly worse in women than in men (50.9% versus 70.8%; P=0.007), but the specificities were similar (84.1% versus 81.3%; P=0.48). The sensitivity in both the female and male groups was significantly higher with CMR than SPECT (P<0.0001 for both), but the specificity was similar (P=0.77 and P=1.00, respectively). For perfusion-only components, CMR outperformed SPECT in women (area under the curve, 0.90 versus 0.67; P<0.0001) and in men (area under the curve, 0.89 versus 0.74; P<0.0001). Diagnostic accuracy was similar in both sexes with perfusion CMR (P=1.00) but was significantly worse in women with SPECT (P<0.0001) (63).

In cardiac amyloidosis (CA), LGE-CMR showed a sensitivity of 95% and a specificity of 98% for the diagnosis of CA. The combination of a characteristic LGE pattern indicating CA with unremarkable monoclonal protein studies resulted in the diagnosis of ATTR-CA (confirmed by EMB) with a specificity of 98% [95% confidence interval (CI) 92–100%] and a positive predictive value (PPV) of 99% (95% CI 92–100%), respectively, while the EMB risk of complications was 3.13%, but without any detrimental or persistent complications (64).

Finally, a study assessing the diagnostic performance of CMR, compared with endomyocardial biopsy (ENB) in patients with suspected acute myocarditis (AMC) and chronic myocarditis (CMC) showed that the best diagnostic performance was observed in patients with suspected AMC (sensitivity, 81%; specificity, 71%; and accuracy, 79%). In contrast in suspected CMC, CMR was found to be unsatisfactory (sensitivity, 63%; specificity, 40%; and accuracy, 52%) (65).

## CMR in Diabetes: Dreams and reality

The 2019 ESC Guidelines on diabetes, pre-diabetes and cardiovascular diseases developed in collaboration with the EASD (66) support that:

1. Resting ECG is recommended in patients with DM with hypertension or suspected CVD
2. Carotid or femoral ultrasound should be considered for plaque detection as CV risk modifier
3. Screening for CAD with coronary CT angiography and functional imaging may be considered,
4. Coronary artery calcium (CAC) scoring may be considered as risk modifier,
5. Ancle-branchial index (ABI) may be considered as risk modifier,
6. Carotid ultrasound intima-media thickness for CV risk is not recommended.

It is really impressive that this recent document does not recommend echocardiography, the cornerstone cardiovascular imaging modality, as a screening tool to detect early myocardial changes in DM. However, adolescents with T1D exhibit early changes in blood pressure, peripheral vascular function, and left ventricular myocardial deformation indices with a shift from longitudinal to circumferential shortening (67). Additionally, the evaluation of myocardial mechanics at rest and during dobutamine echo stress showed that T2DM patients presented with altered global diastolic function but preserved systolic function. Furthermore, deformation imaging indexes were similar between groups at rest, but significant differences were noticed under dobutamine infusion for longitudinal strain (68).

**Figure 7:**
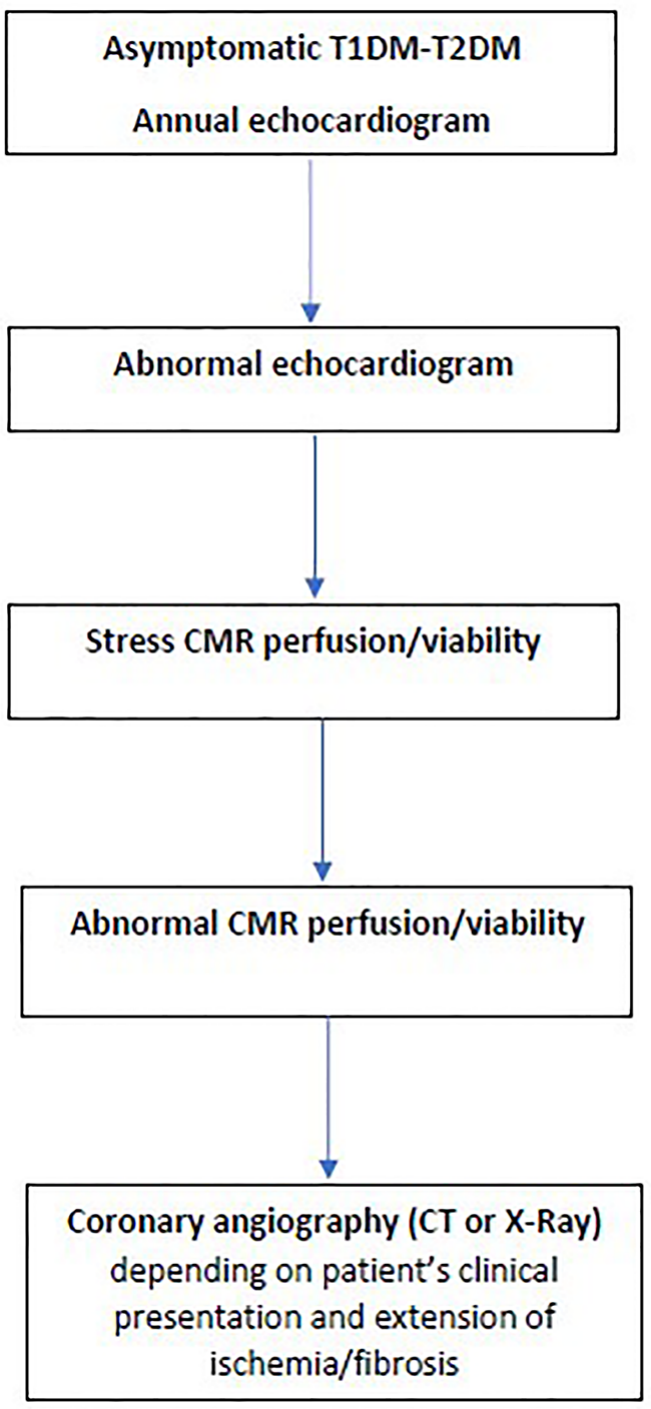
Suggestive algorithm for the evaluation of CAD in DM.

It is out of the scope of this article to analyze the role of all recommended imaging modalities in the evaluation of cardiac involvement in DM. However, a glimpse to the literature showed that ECG has low partial sensitivity and specificity for predicting coronary artery stenosis with accuracy ranged 58.5% to 62.0% based on coronary artery analysis (69). Furthermore, CT coronary angiography, although it has excellent sensitivity/specificity, is an ionizing radiation modality and therefore, it cannot be recommended as an annual screening tool (70). Therefore, before expensive, sophisticated modalities will be proposed as screening tools for CAD in DM, we should use the experience coming from the evaluation of other cardiovascular diseases. According to this experience, echocardiography including also the new indices should be incorporated in the annual routine evaluation of DM patients, because it is low cost, easily available, with great expertise between cardiologists and can detect pre-clinical changes in both T1DM and T2DM (67, 68). Furthermore, an imaging algorithm including all steps after the echocardiographic evaluation should be proposed (**Figure 7**). CMR, although it is the ideal imaging modality for CAD detection, it cannot be proposed as a screening tool for all DM patients, due to various reasons mainly including low availability, high expertise, and increased cost. However, there are clear indications for CMR in DM including:

1. Stress CMR perfusion, viability assessment, because it can detect lesions missed by both echocardiography or SPECT;
2. Evaluation of heart failure and arrhythmia in DM patients, because it can reveal the pathophysiologic background behind these entities and guide further treatment;
3. Assessment of ischemia/fibrosis burden in DM patients with known CAD in order to make to final decision regarding re-vascularization or heart transplantation;
4. Inconclusive echocardiographic evaluation, due to technical reasons, such as obesity or obstructive lung diseases;
5. Doubtful echocardiographic results that need further evaluation, such as differential diagnosis between diabetic cardiomyopathy and heart failure, due to coronary artery disease.

## Conclusions

The multifaceted nature of CMR allows the assessment of ventricular function/remodeling, myocardial oedema, ischemia during stress, and symptomatic or asymptomatic myocardial replacement/diffuse fibrosis. With increasing availability and easier acquisition/processing, CMR will rapidly become more widely available to the clinicians for early detection of CVD in DM, specifically in subclinical diabetic heart disease.

## Author Contributions

SM, FB, and GM-M contributed to conception and design of the study. SM wrote the first draft of the manuscript. FB, GM-M, AG, OK, VV, GK, EC, and GC wrote sections of the manuscript. All authors contributed to the article and approved the submitted version.

## Disclaimer

The authors are responsible for the choice and presentation of views contained in this article and for opinions expressed therein, which are not necessarily those of UNESCO and do not commit the Organization.

## Conflict of Interest

The authors declare that the research was conducted in the absence of any commercial or financial relationships that could be construed as a potential conflict of interest.

The reviewer AM-F declared a shared affiliation with some of the authors, SM, FB, EC, and GC, to the handling editor at time of review.
